# Innovative Pathogen Reduction in Exported Sea Bass Through Atmospheric Cold Plasma Technology

**DOI:** 10.3390/foods13203290

**Published:** 2024-10-17

**Authors:** Şehnaz Yasemin Tosun, Sehban Kartal, Tamer Akan, Sühendan Mol, Serap Coşansu, Didem Üçok, Şafak Ulusoy, Hande Doğruyol, Kamil Bostan

**Affiliations:** 1 Department of Seafood Processing Technology, Faculty of Aquatic Sciences, Istanbul University, Kalenderhane, Onaltı Mart Şehitleri St., No. 2, Vezneciler-Fatih, 34134 Istanbul, Türkiye; yasemin@istanbul.edu.tr (Ş.Y.T.); suhendan@istanbul.edu.tr (S.M.); ducok@istanbul.edu.tr (D.Ü.); safak@istanbul.edu.tr (Ş.U.); 2 Department of Physics, Faculty of Science, Istanbul University, Vezneciler, 34452 Istanbul, Türkiye; sehban@istanbul.edu.tr; 3 Department of Physics, Faculty of Science, Eskişehir Osmangazi University, 26040 Eskişehir, Türkiye; akan@ogu.edu.tr; 4 Department of Food Engineering, Engineering Faculty, Sakarya University, 54050 Sakarya, Türkiye; 5 Department of Food Safety, Faculty of Aquatic Sciences, Istanbul University, Fatih, 34134 Istanbul, Türkiye; dogruyol@istanbul.edu.tr; 6 Department of Gastronomy and Culinary Arts, Faculty of Fine Arts, Istanbul Aydın University, Küçükçekmece, 34295 Istanbul, Türkiye; kamilbostan@aydin.edu.tr

**Keywords:** seafood, fish, food safety, health risk, emerging technology

## Abstract

The safety of sea bass is critical for the global food trade. This study evaluated the effectiveness of atmospheric cold plasma in reducing food safety risks posed by *Salmonella* Enteritidis and *Listeria monocytogenes*, which can contaminate sea bass post harvest. Cold plasma was applied to inoculated sea bass for 2 to 18 min, achieving a maximum reduction of 1.43 log CFU/g for *S.* Enteritidis and 0.80 log CFU/g for *L. monocytogenes* at 18 min. Longer treatments resulted in greater reductions; however, odor and taste quality declined to a below average quality in samples treated for 12 min or longer. Plasma treatment did not significantly alter the color, texture, or water activity (aw) of the fish. Higher levels of thiobarbituric acid reactive substances (TBARSs) were observed with increased exposure times. Cold plasma was also tested in vitro on *S.* Enteritidis and *L. monocytogenes* on agar surfaces. A 4 min treatment eliminated the initial loads of *S.* Enteritidis (2.71 log CFU) and *L. monocytogenes* (2.98 log CFU). The findings highlight the potential of cold plasma in enhancing the safety of naturally contaminated fish. Cold plasma represents a promising technology for improving food safety in the global fish trade and continues to be a significant area of research in food science.

## 1. Introduction

Fish is generally considered a safe food. However, it may be contaminated with bacterial pathogens and may cause outbreaks when eaten raw or undercooked [1]. Fish provides an ideal environment for microbial growth due to its high water content [2]. Additionally, the risk of contamination arising from improper conditions throughout the supply chain creates a vulnerability in fish safety [3].

*Salmonella* Enteritidis and *Listeria monocytogenes* have been reported to be among the most important pathogens that may contaminate fish and other seafood, as they can cause Salmonellosis and Listeriosis [4]. These pathogens have been implicated in increasing fish recalls in both domestic and imported markets [1]. *Salmonella* is reported to be the leading bacterial cause of outbreaks associated with fish consumption, and aquaculture fish contaminated with this pathogen have been documented in many countries [5]. Heinitz et al. [6] studied the presence of *Salmonella* in imported and domestic seafood and emphasized the importance of innovative practices to reduce its incidence in fish. *L. monocytogenes*, on the other hand, is the most common bacterial cause of recalls for fish and fish products despite its lower incidence [1] and high mortality rate [7]. This pathogen may be transferred to fish by cross-contamination after harvest and can grow during distribution and storage due to its psychrotrophic properties [8]. As Listeriosis is a food safety issue affecting many countries worldwide, it is essential to monitor the presence of *L. monocytogenes* in fish and eliminate, reduce, or inhibit its growth [9].

Today, the most important fish farmed commercially in the Mediterranean is the European sea bass, and Türkiye is the largest producer and exporter of this species in Europe. Market conditions in Türkiye directly affect the European fish market [10]. Almost all non-EU sea bass comes from Türkiye, which has increased the share of Turkish sea bass in European fish consumption [11]. Pathogenic bacteria in cultured fish are mostly related to improper cultivation methods, surrounding water habitats, and unhygienic post-harvest handling conditions [8,12].

The food safety risks posed by the pathogens have created a demand for new methods to reduce contamination without changing the fresh properties of the food and leaving any harmful residues [13]. Although traditional preservation methods can inactivate pathogens, they also change the sensory properties of food; therefore, there is a significant need to focus on non-thermal techniques that can be used to reduce contamination [14]. Advances in food science and technology have led to the development of new methods, such as cold plasma, to address the challenges of ensuring quality and safety in fish. Research must focus on technological improvements for sustainable growth in the seafood industry. Cold plasma technology is promising due to low energy consumption, operational efficiency, and alignment with consumer preferences and demands [15]. In recent years, cold plasma has gained attention as a process that does not leave residue on the product, does not create heat, contains no chemicals, and is environmentally friendly [2]. Since cold plasma is applied at low temperatures, it promises a method of preservation that may be particularly suitable for heat-sensitive materials such as fish [16].

When a gas is energized, the plasma is generated with unique properties, including antibacterial effects [13]. Plasma is a rich source of free radicals, electrons, excited species, ions, anions, cations, and ultraviolet photons [17]. Although there is no definitive statement on the inhibitory effect of plasma, the main factors reported have been the damage to the surfaces of bacterial cells by reactive species, photodesorption of ultraviolet photons, devastation of genetic material, diffusion of reactive species, and oxidation of macromolecules [16]. A recent study investigated the inactivation kinetics of cold plasma on *S.* Enteritidis and *L. monocytogenes*, stating that the primary effect was the disruption of normal aerobic respiration, leading to cell death [18]. Parameters such as the design of the plasma generating system, carrier gas, current density, voltage, and frequency directly contribute to the efficiency of cold plasma [13]. Although many systems produce plasma using inert gasses, atmospheric cold plasma has attracted interest due to its low cost and ease of application [14]. Since the food matrix and various operating parameters of different plasma systems affect the level of inhibition achieved [19], further studies are needed to implement this technology in the fish industry [20,21].

To our knowledge, the effect of atmospheric pressure cold plasma on *S.* Enteritidis and *L. monocytogenes* in fresh fish has not been investigated, and the changes in quality properties due to this treatment have not been comprehensively evaluated. Therefore, this study aimed to (1) investigate the effect of various exposure times of atmospheric cold plasma on reducing *S.* Enteritidis and *L. monocytogenes* inoculated onto sea bass and (2) determine the impact of cold plasma treatment on the quality attributes of raw fresh sea bass filets. Considering that the effect of cold plasma may vary depending on the structure of the bacterial cell, both a Gram-negative (*S.* Enteritidis) and a Gram-positive (*L. monocytogenes*) pathogen were studied.

## 2. Materials and Methods

### 2.1. Atmospheric Pressure Cold Plasma System

**Figure 1:**
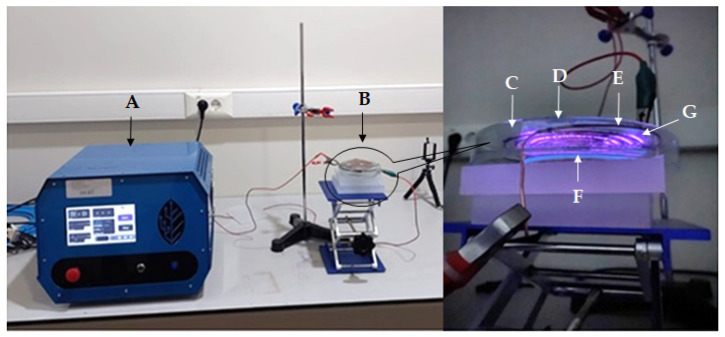
Original atmospheric cold plasma equipment ((A) power supply; (B) plasma generation cite; (C) glass Petri dish lid; (D) copper plate; (E) copper wire; (F) sample site; (G) cold plasma).

In this study, an original atmospheric pressure cold plasma generating system was designed. The system produces surface dielectric barrier discharge (SDBD) plasma. A copper plate (9 × 11 cm) was fixed to the outer surface of a 14 cm diameter glass Petri dish lid, and a copper wire with a 1.5 mm diameter and 90 cm long was fixed to the inner surface. The copper wire was fixed spiral to the inner surface of the lid, from the center outward. In plasma generation, the inner electrode was connected to the high voltage of the 50 kV-2500 Hz alternative current (AC) power supply (Figure 1). The outer electrode was connected to the ground. Experiments were conducted using a total power range of 25–360 W. The apparatus was run under normal atmospheric conditions, using atmospheric air as the process gas. The temperature of the copper electrode was measured using a K-type thermocouple, and it stayed consistently below 28 °C for all treatments. When the system was energized, purple-colored atmospheric cold plasma was generated between the glass Petri dish lid and spiral wire.

### 2.2. Effect of Plasma Treatment on Pathogens

#### 2.2.1. Preparation of the Bacterial Inoculums

Stock cultures of *Salmonella enterica* serovar Enteritidis ATCC 13076 and *Listeria monocytogenes* ATCC 7644 were obtained from the Department of Food Engineering at Sakarya University and stored at −40 °C. The stock cultures were activated twice in Tryptic Soy Broth (TSB; Merck, Darmstadt, Germany) for *S.* Enteritidis and TSB supplemented with 0.6% yeast extract for *L. monocytogenes* by incubating at 37 °C for 18–24 h. The TSB cultures were centrifuged at 3900 rpm for 10 min (EBA 20 Hettich, Tuttlingen, Germany). After centrifugation, the supernatant was discarded, and the resulting pellets were washed twice with 0.1% peptone water. Finally, the pellets were suspended in 10 mL of 0.1% sterile peptone water to prepare the inoculum with a 10^5^–10^6^ CFU/mL cell concentration.

#### 2.2.2. In Vitro Plasma Treatment

The antimicrobial activity of cold plasma against both pathogens on the agar surface was tested. Serial dilutions of each pathogen were prepared, and 0.1 mL diluted inoculums of *S.* Enteritidis and *L. monocytogenes* were spread onto supplemented Xylose-Lysine-Tergitol 4 (Merck, Darmstadt, Germany) and supplemented Palcam Listeria Selective Agar (Merck, Darmstadt, Germany), respectively. Then, the dishes were exposed to cold plasma for 0, 2, 4, 6, 8, 10, 12, 14, 16, and 18 min. Samples designated and coded as “0 min” were the control samples not treated with plasma. The distance between the plasma source and the agar surface was maintained at 2 cm during treatment. Each medium was incubated at the appropriate time and temperature (at 37 °C for 24–48 h for *S.* Enteritidis and at 35 °C 48 h for *L. monocytogenes*). After the incubation period, the colonies were counted, and the results were expressed as log CFU. The reduction was calculated by subtracting the number of pathogens remaining in the plasma-treated Petri dishes from the untreated ones.

#### 2.2.3. Inoculation of Fish Samples and Plasma Treatment

Farmed sea bass (*Dicentrarchus labrax*, L. 1758) were purchased from an aquaculture firm in the Aegean Sea and delivered to the laboratory in foam boxes under cold chain conditions within 18 h. Samples (10 g) were taken from the dorsal part of the sea bass, and the skin was removed. A 0.1 mL portion of the diluted inoculum of *S.* Enteritidis or *L. monocytogenes* was applied to the sample surface and spread with a Drigalski spatula targeting an inoculation level of 4–5 log CFU/g. After inoculation, the samples were kept in a laminar airflow cabinet (CRYSTE, Bucheon, Republic of Korea) for 15 min to allow bacterial attachment. Then, the inoculated fish samples were exposed to cold plasma for 0, 2, 4, 6, 8, 10, 12, 14, 16, and 18 min. Following plasma treatments, each fish sample (10 g) was mixed with 90 mL of Maximum Recovery Diluent (HIMEDIA, Thane, India) and homogenized separately using a stomacher (IUL Instruments, Barcelona, Spain). Serial dilutions (1:10 diluent) were then prepared and spread onto selective agars appropriate for the inoculated pathogens. After incubation, bacterial colonies were counted and expressed as log CFU/g.

### 2.3. Effect of Plasma Treatment on Quality Characteristics of Sea Bass

To evaluate the effect of cold plasma on the quality characteristics of sea bass, uninoculated fish samples were exposed to cold plasma for the same durations, and sensory, TBARS, color, texture, and water activity (aw) analyses were performed.

#### 2.3.1. Sensory Analysis

Ten panelists analyzed the sensory properties of plasma-treated and untreated sea bass samples. Uninoculated fish samples were used for sensory analyses. The pathogen-free samples were exposed to plasma for 0, 2, 4, 6, 8, 10, 12, 14, 16, and 18 min, the same as the inoculated samples, and then subjected to sensory evaluation. The panelists, academics from the Faculty of Aquatic Sciences, were previously trained in assessing seafood quality and were experienced in sensory analysis. The panelists were selected and trained based on their perception of basic tastes during the training. Sensory evaluation was performed using a nine-point hedonic scale to assess appearance, texture, odor, taste, and overall quality (1: extremely poor, 2: very poor, 3: poor, 4: below average, 5: average, 6: above average, 7: good, 8: very good, 9: excellent). Appearance, texture, odor, and overall quality were assessed on raw samples, while taste was evaluated immediately after cooking. Fish samples were randomly coded with three-digit numbers and evaluated by panelists in a well-ventilated sensory analysis laboratory with appropriate lighting. Panelists were prevented from interacting during the evaluation. For odor assessment, samples were presented in covered Petri dishes, and the evaluation was conducted by slightly opening the lid. Taste was assessed in fish samples cooked in a microwave oven in a glass Petri dish [14,22]. During the taste analysis of cooked samples, panelists were given drinking water to cleanse their palate between tastings. The texture quality of the fish was evaluated by finger touch, considering hardness, softness, and elasticity.

#### 2.3.2. Thiobarbituric Acid Reactive Substance (TBARS) Analysis

The TBARS was determined using the method proposed by Kanwate et al. [23]. Homogenized fish (5 g) was distilled by heating with 2 mL of 4N HCl (Merck, Darmstadt, Germany) and distilled water. The resulting distillate (5 mL) was mixed with 5 mL TBA reagent (Merck, Darmstadt, Germany) and boiled for 30 min in a water bath. Absorbance was measured at 532 nm, and the Malondialdehyde (MDA) concentration was expressed as MDA/kg fish sample.

#### 2.3.3. Color Measurements

The color was measured using a Chroma Meter (CR 400/410, Konica Minolta, Osaka, Japan). To determine the color difference (Δ*E*) between untreated samples and plasma-treated ones, the following equation was used [24]:

$$∆E=\sqrt{{\left(∆L\right)}^{2}+{\left(∆a\right)}^{2}+{\left(∆b\right)}^{2}}$$

#### 2.3.4. Texture Profile Analysis (TPA) and Water Activity

The texture profile of fish samples was analyzed using a Brookfield CT3-1500 Texture Analyzer (Brookfield Engineering Laboratories, Middleboro, MA, USA) with a load cell capacity of 1.5 kg. The analysis was conducted at room temperature, and 3 × 3 cm^2^ fish pieces were used. Texture profile analysis was performed using a compression test. The working parameters during texture analysis were as follows: target value: 2.000 N, holding time: 5 s, trigger load: 0.020 N, and test speed: 1.00 mm/s. The TA 50 probe was used.

The water activity (aw) values of fish samples were measured using a water activity meter (HC2-S-AW, Rotronic AG, Bassersdorf, Switzerland) at ambient temperature (20 °C).

### 2.4. Statistical Analyses

Microbiological, chemical, and instrumental analyses were performed in triplicate. The IBM SPSS Statistics 20 software program was used to evaluate all data statistically. The data showed a normal distribution. A one-way ANOVA test was conducted to determine the difference between the groups. Tukey’s or Tamhane’s tests were performed according to the homogeneity of variances. The significance threshold was *p* < 0.05.

## 3. Results and Discussion

### 3.1. In Vitro Inactivation of S. Enteritidis and L. monocytogenes

**Figure 2:**
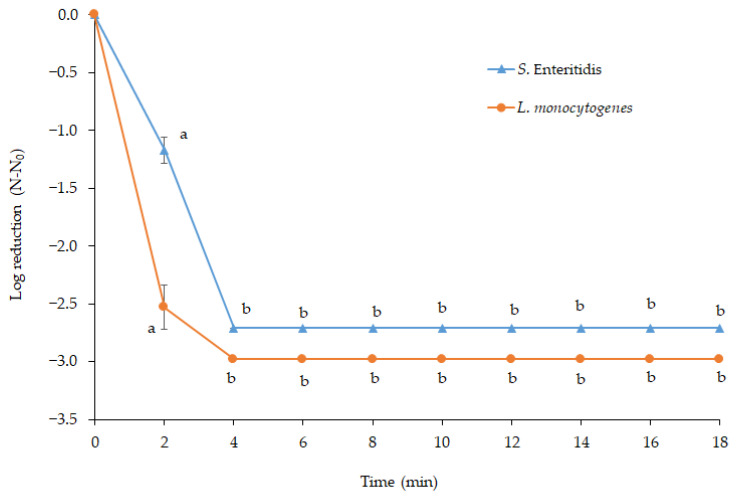
In vitro reduction in *S.* Enteritidis and *L. monocytogenes* by atmospheric cold plasma (a, b: different letters show significant differences between reduction rates, *p* < 0.05).

Cold plasma was applied to *S.* Enteritidis and *L. monocytogenes* cells inoculated on solid media to determine its in vitro effects. The initial loads of *S.* Enteritidis and *L. monocytogenes* were 2.71 and 2.98 log CFU. A four-minute plasma exposure was sufficient to completely inhibit the initial loads of both pathogens (Figure 2). Plasma discharge contains UV radiation and photons and generates reactive species with bactericidal effects [21]. The diffusion of reactive species, intrinsic photodesorption of UV photons, etching of the cell surface, and oxidation, volatilization, and destruction of genetic material are the main factors behind the bactericidal effect. However, the inactivation mechanism is still not fully understood [16]. The previous studies conducted on agar surfaces or in liquid bacterial suspensions to inactivate *Salmonella* serovars and *L. monocytogenes* via cold plasma treatment reported reductions of up to 8 log CFU depending on the gas compositions, treatment time, treatment distance, etc. Kayes et al. [25] reported a 3.7 log reduction in *S.* Enteritidis colonies on tryptic soy agar after a one-minute air plasma treatment. Lee et al. [26] conducted a study on the in vitro reduction in *L. monocytogenes* by cold plasma, achieving a reduction ranging from 0.87 to 7.59 log units after 2 min of treatment, with the highest reduction obtained using a N₂ + O₂ combination. When *Salmonella typhimurium* was exposed to a plasma jet on agar plates for 5 and 10 min from different treatment distances, bacterial colonies were reduced by 0.87–1.70 and 2.38–3.77 log CFU, respectively [27]. Yong et al. [28] determined complete inactivation after 1 and 10 min of plasma treatment in *S. typhimurium* and *L. monocytogenes* with initial counts of 8 log CFU. In a recent study by Qian et al. [29], the liquid suspensions of *S.* Enteritidis and *L. monocytogenes* were treated with air plasma for 2.5 min, and seven log reductions were recorded for both pathogens.

The reduction rate in *L. monocytogenes* was higher than that of *S.* Enteritidis after a 2 min plasma treatment (Figure 2), indicating that *L. monocytogenes* was more sensitive to cold plasma at the beginning of the treatment. Nevertheless, after a 4 min plasma treatment, no viable cells of both pathogens were detected. The type of cell wall appears to have a significant role in the sensitivity of bacterial cells against cold plasma. The outer membrane of Gram-negative bacteria is more sensitive to plasma-reactive species than the cell walls of Gram-positive bacteria. In contrast, the tick cell walls of Gram-positives prevent the entry of these reactive species into the cell [30]. The inactivation mechanisms differ between Gram-negative and Gram-positive bacteria. Cold plasma treatment induces the formation of active species that attack the outer membrane of Gram-negative bacteria. The phospholipids and lipopolysaccharides, the outer membrane’s main components, are exposed to lipid peroxidation. In addition, porins on the outer membrane facilitate the penetration of reactive species, ultimately destroying the outer membrane and thin cell wall (peptidoglycan layer), which results in the leaching of intracellular components and oxidation of DNA and lipids [31]. For Gram-positive bacteria, on the other hand, intracellular components are the primary targets causing cell wall or membrane leakage. However, the cell walls of Gram-positive bacteria are less injured than those of Gram-negative bacteria [32]. Some studies conducted in in vitro conditions, i.e., on agar surface or liquid bacterial suspensions, have reported higher resistance of Gram-positives than Gram-negatives [28,29,32,33]. Conversely, according to Lu et al. [34] and Nishime et al. [35], Gram-negative bacteria tend to exhibit greater resistance. Moreover, Colejo et al. [36] reported that the plasma treatment times required for a 99% reduction in *S.* Enteritidis and *L. monocytogenes* populations on Brain Heart Infusion (BHI) Agar were 2.38 and 0.42 min, respectively. These varying results may stem from other factors, such as process conditions, relative humidity, treatment time, etc., in addition to cell wall type [30,31].

### 3.2. Effect of Cold Plasma on S. Enteritidis and L. monocytogenes in Sea Bass

**Figure 3:**
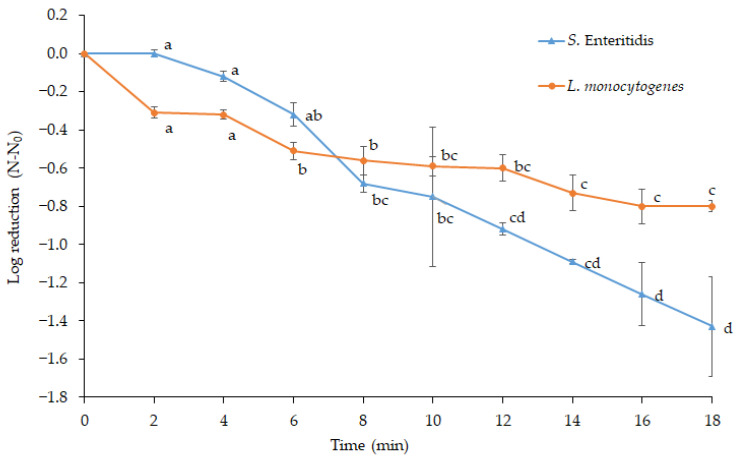
Reduction in *S.* Enteritidis and *L. monocytogenes* on sea bass by atmospheric cold plasma (a–d: different letters show significant differences in reduction rates, *p* < 0.05).

Table 1 and Figure 3 depict the counts of survivors and reduction rates, respectively, in cold plasma-treated sea bass as a function of treatment time. The total reductions in *L. monocytogenes* and *S.* Enteritidis counts were 0.80 and 1.43 CFU/g after 18 min of cold plasma treatment (*p* < 0.05). Based on previous studies, it may be speculated that cold plasma treatment produces similar levels of pathogen reduction in muscle foods. Gök et al. [37] exposed dry-cured beef to atmospheric cold plasma created with argon, oxygen, or their mixtures and achieved a maximum reduction of 0.83 log CFU/cm^2^ in the number of *L. monocytogenes*. Likewise, in-pack cold plasma generated by argon and oxygen mixture reduced *Listeria innocua* by 0.8–1.6 log CFU/g in Bresaola [38]. Kim et al. [39] treated pork filets with He and He + O_2_ cold plasma for up to 10 min, achieving less than 1 log reduction in *L. monocytogenes* counts. A corona plasma jet was reported to reduce the number of *L. monocytogenes* in pork by about 1 log cycle [40]. The maximum reductions in *S. typhimurium* and *L. monocytogenes* in ham treated with cold plasma for 20 min were 1.14 and 1.02 log CFU, respectively [41]. Roh et al. [42] reported a 1.7 log reduction in S*. typhimurium* in the boiled chicken breast by in-package atmospheric dielectric barrier discharge cold plasma treatment. A later study reported a 0.9–1.0 log CFU/g reduction in *Salmonella* sp. and *L. monocytogenes* in chicken breast with a similar in-package cold plasma treatment [43]. Similarly, Zhuang et al. [44] reported that in-package dielectric barrier discharge cold plasma treatment reduced *Salmonella* in chicken breasts by up to 1 log. Therefore, the reduction rates observed in the current study align with those reported in previous studies involving muscle foods.

A higher reduction was achieved in *S.* Enteritidis than in *L. monocytogenes* after an 18 min plasma treatment. Similarly, Colejo et al. [36] determined that 8 min cold plasma provided a 0.49 log reduction in *L. monocytogenes* and a 0.70 log reduction in *S.* Enteritidis population inoculated on smoked salmon slices. An in-pack dielectric barrier discharge plasma for 10 min was reported to reduce *Salmonella typhimurium* and *L. monocytogenes* in raw chicken breast by 2.71 and 2.14 log CFU/g, respectively [45]. In addition, Yong et al. [28] reported 2.26 and 3.11 log CFU/g reductions in *L. monocytogenes* and *S. typhimurium* on cheese slices following cold plasma treatment. As mentioned before, the differing sensitivities of the two pathogens may be attributed to structural differences in their cell walls.

The reduction rates of both pathogens on the surface of sea bass were lower than those on the agar surface. A two-minute plasma treatment reduced *L. monocytogenes* and *S.* Enteritidis on the agar surface by 2.53 and 1.17 log CFU, respectively (Figure 2). However, the equal treatment of sea bass only yielded a reduction of 0.31 log CFU in *L. monocytogenes* and no reduction in *S.* Enteritidis counts. Likewise, Colejo et al. [36] reported approximately 2 and 3.3 log CFU reductions in *S.* Enteritidis and *L. monocytogenes* inoculated on BHI Agar after a 2 min plasma treatment. In contrast, an identical cold plasma treatment caused only 0.22 and 0.20 log CFU reductions on smoked salmon, respectively. They extended the treatment time to 15 min, yet the reduction rates did not exceed the 0.6–1.2 log cycles. Moreover, Yong et al. [28] found that cold plasma was more effective in inactivating bacteria on agar plates than cheese slices.

A possible reason for the lower antimicrobial activity of cold plasma on sea bass compared to agar plate may be the surface roughness of fish. The crevices and fissures on the fish surface may act as shelters for protecting bacterial cells from the reactive species generated during cold plasma treatment [30]. Therefore, the surface topography significantly determines the microbial inactivation rate [36]. For example, Ziuzina et al. [46] reported better bacteria inactivation on the smooth surface of tomatoes than on strawberries. The food matrix generally comprises proteins, lipids, and carbohydrates. When evaluating the effectiveness of antimicrobial treatments on foods, the interaction of the food matrix, particularly with proteins and lipids, should be considered [47]. In this study, the presence of proteins and fats in fish flesh may be another factor contributing to the varying levels of inactivation compared to the in vitro environment. The other possible factors affecting the inactivation efficacy of cold plasma are relative humidity, water content, and initial microbial count [30,48]. The chemical composition of the food, including the presence of organic materials, can interact with plasma-generated reactive species, affecting the overall antimicrobial activity. Bacterial internalization through natural openings or damaged sites in the produce can protect microorganisms from plasma treatment, making it less effective.

Additionally, the nutrient content of the food can influence the survival and growth of microorganisms, subsequently affecting the efficacy of cold plasma treatment. Relative humidity (RH) also plays a critical role in achieving effective microbiological control, and higher moisture content can enhance inactivation efficiency due to the presence of reactive oxygen species (ROS) generated in the discharge emission spectra [17]. Therefore, the findings in this study, along with the previous literature, reveal that the properties of food should be considered comprehensively, in addition to the processing conditions in cold plasma studies.

The reduction in *L. monocytogenes* on sea bass with prolonged plasma application times showed no further decrease after 14 min (Figure 3). Such a tailing effect in the survival curves of *L. monocytogenes* was reported previously [29,36,49,50]. This tailing effect may be attributed to non-homogeneous populations containing plasma-resistant cells [48] or the protective actions of certain cellular metabolites [36]. Moreover, exterior cells can shield interior cells from the reactive species generated by cold plasma in a cell aggregate, especially at high microbial loads [30].

The contamination of fish with pathogens after harvest is a significant food safety risk that threatens public health. Although the contamination of fish is usually at very low levels, it can still cause diseases that carry the risk of death. The Food and Agriculture Organization emphasizes that *Salmonella* sp. should be completely absent from fish meat [51]. *L. monocytogenes* is the most common cause of fish and fish product recalls because of its presence in processing environments and ability to multiply at cold temperatures, making it a significant concern for ready-to-eat fish products [1]. Despite the high prevalence of *L. monocytogenes*, the risk assessments indicate that its contamination level is generally low [52]. In this study, the inoculation levels of pathogens were 4 log CFU/g, exceeding typical contamination levels. Therefore, given that these pathogens typically contaminate raw fish at very low levels, the reduction rates of 0.80–1.43 log CFU/g achieved through plasma treatment in this study could significantly mitigate hazards and enhance the microbial safety of fish. Since marine fish offered for consumption may be contaminated with pathogenic bacteria, implementing risk reduction practices is essential to prevent foodborne illness [53]. Ensuring food safety is crucial for minimizing food loss and waste. In fisheries, maintaining food safety is key to meeting the Sustainable Development Goals (SDGs). Fish can be exposed to pathogens in their habitats, and practices related to harvesting, handling, and storage may introduce further contamination risks, thereby compromising safety [54]. Developing and implementing intervention strategies to reduce or eliminate pathogens in fish after capture or harvest is crucial for ensuring food safety. The practical significance of pathogen reduction lies in its ability to minimize the risk of foodborne illnesses, protect consumer health, and maintain the integrity of the food supply. There is a growing preference for environmentally friendly and energy-efficient practices to reduce pathogenic bacteria counts. In this context, non-thermal methods have become effective alternatives for pathogen control while maintaining product quality and sustainability [1].

The primary challenges of cold plasma technology include determining the efficacy of various plasma systems on different food commodities and the need for extensive data during the regulatory approval process [55]. However, the environmentally friendly nature, cost-effectiveness, and ease of application of cold plasma technology present significant advantages regarding its industrial potential and integration into existing processing lines. There is an urgent need for active research to facilitate the upscaling of commercial applications [56]. The applicability of cold plasma to fresh, processed, and packaged foods enhances its potential for use. Integrating conveyor belts into existing processing lines to transport fish through the cold plasma-generated area may present new challenges for industrial applications.

### 3.3. Effect of Cold Plasma on Sensory Properties of Fresh Sea Bass

**Figure 4:**
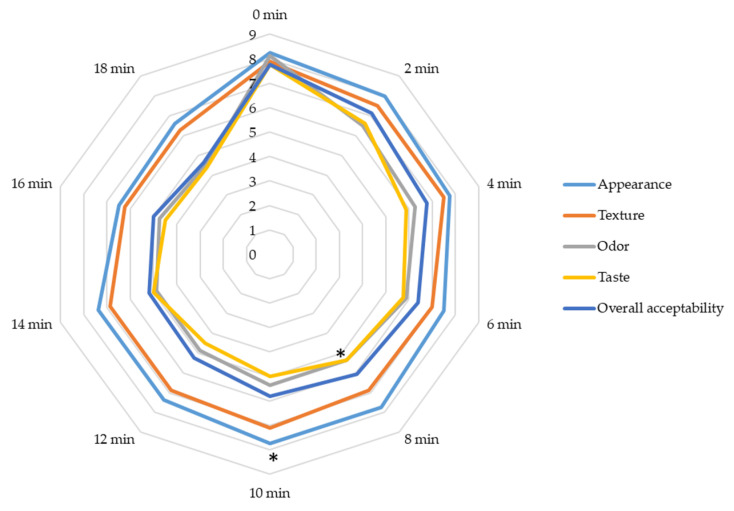
Sensory analysis of sea bass treated with atmospheric cold plasma for various durations (* decreases in odor and taste after 8 min and in overall acceptability after 10 min are significant, *p* < 0.05).

In this study, sensory analysis was performed on pathogen-free samples to identify the optimal cold plasma application time that effectively reduces pathogens without having notable effects on sensory properties. Plasma applications of up to 10 min maintained the appearance, texture, odor, and overall quality characteristics of the fish above 5 points (Figure 4). However, when cold plasma was applied for 12 min or longer, panelists detected an ozone odor, resulting in odor scores falling below 5 points. Additionally, taste scores also dropped below average quality after 12 min of plasma application. The sensory parameters of plasma-treated samples generally showed no differences (*p* > 0.05), except for decreases in odor and taste after 8 min (*p* < 0.05) and in overall acceptability after 10 min (*p* < 0.05). Yong et al. [57] used an in-pack flexible thin-layer plasma to reduce bacteria in beef jerky. They reported decreased taste, odor, and general acceptability values with increased plasma treatment times, similar to our findings. Likewise, Olatunde et al. [58] exposed Asian sea bass slices to cold plasma generated in a mixture of Ar, CO_2_, and O_2_, reporting no changes in appearance, color, or texture characteristics but observed reduced values for odor, taste, and overall acceptability. In another study, the negative effects of cold plasma on the sensory properties of pork were highlighted, emphasizing the necessity to explore cold plasma applications that can effectively inactivate pathogens without compromising sensory acceptance. It has been noted that the sensory effects of cold plasma may vary depending on the type of food [39]. Reports indicate that in-package dielectric barrier discharge plasma negatively impacts the taste values of pork and beef. The decreases in taste and odor scores are primarily attributed to free radicals generated during the plasma process, leading to fat and protein oxidation and secondary oxidation products [14]. Bourke et al. [59] highlighted that meat products are particularly susceptible to lipid peroxidation due to their lipid content, noting that applying plasma to fish poses challenges in maintaining quality. This study revealed that cold plasma applications for up to 12 min effectively reduced *S.* Enteritidis and *L. monocytogenes* without significantly affecting sensory acceptability. This result highlights the industrial potential of cold plasma as a method that can achieve pathogen reduction while preserving the fresh qualities of the fish. Given that the sensory impacts of cold plasma produced by different devices can vary depending on food types, studies aimed at identifying applications that minimize significant changes in taste and odor are crucial for commercializing this technology [60].

### 3.4. Effect of Cold Plasma on Aw and Texture of Fresh Sea Bass

Since plasma treatment may cause desiccation in food [61], the water activity (aw) of sea bass samples was measured in this study. The aw value of 0.96 in fresh sea bass did not show a significant change (*p* > 0.05) due to atmospheric cold plasma treatments for up to 18 min. Lee et al. [62] treated fish balls with cold plasma in a shorter time (3 min) and reported no change in the aw value. In the current study, the fact that cold plasma treatment for up to 18 min did not change the aw values indicates treatment’s potential for application.

Texture profile analysis was conducted to assess the potential effects of plasma treatment on the structure of fish. The samples were analyzed for characteristics including hardness1, adhesiveness, resilience, and hardness2. It was determined that different durations of cold plasma treatment did not cause significant changes (*p* > 0.05) in these characteristics of the samples (Table 2). In another study, in-package atmospheric cold plasma was applied to pork and beef for different durations, up to 10 min, for pathogen inactivation. No significant change was reported in the texture properties of both meat types [14]. Additionally, texture parameters of the chicken breast treated with in-package dielectric barrier discharge (DBD) plasma were examined, and the results revealed that both hardness and adhesiveness remained unaffected after up to 10 min of treatment [45]. Furthermore, combining in-pack cold plasma with ZnO was found to preserve the gel properties of fish cakes [62].

On the other hand, De Souza Silva et al. [63] evaluated the shear force of Pacific white shrimp. They reported statistically significant differences before and after 10 min of atmospheric cold plasma application. In our study, the atmospheric cold plasma process did not change the texture parameters significantly (*p* > 0.05), indicating its potential as a preservation method that does not damage fish meat. Further studies involving various food types and processing parameters are needed to validate these findings.

### 3.5. Effect of Cold Plasma on the Color of Fresh Sea Bass

**Figure 5:**
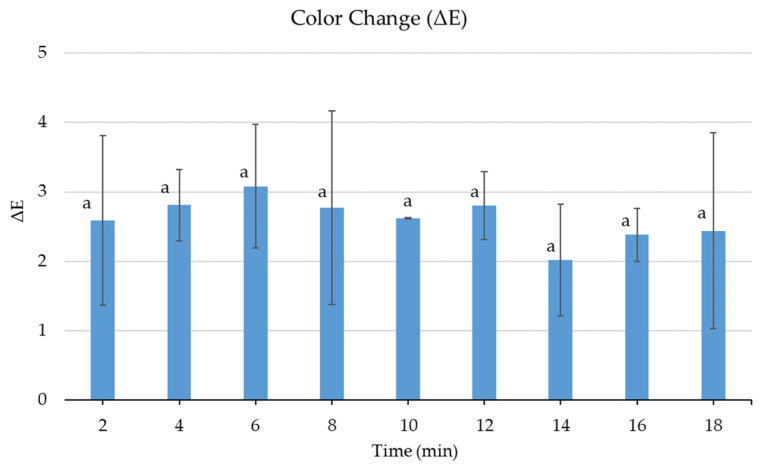
Color change in plasma-treated sea bass compared to untreated samples (a; no significant differences between ΔE vales, *p* > 0.05).

Color is an important factor in food preference and purchase. Therefore, color change, or color difference, is used as an indicator of food acceptability. A detectable difference is deemed perceptible when the ΔE values exceed 3 [64]. In this study, the colors of plasma-treated samples were compared with untreated fish, and it was observed that the color changes were below 3 (Figure 5). The color change in samples treated for 6 min (ΔE = 3.08) slightly exceeded this value. These results show that plasma treatments did not cause a significant color change in sea bass samples (*p* > 0.05).

Similarly, it was reported that in-package cold plasma applied to fish balls in combination with ZnO did not cause a significant color change, and it was stated that the reason for this was that the reactive species formed were not at a level that would cause discoloration when reacted with the product components. Since food color directly affects the consumer, the importance of preventing color change in terms of sales has been emphasized [62]. Likewise, no significant change was reported in the color of the ham treated with cold plasma for up to 3 min [65]. On the other hand, plasma jet treatment for up to 2 min was reported to change the color of pork samples significantly [40]. Since the effect of plasma on food color may differ depending on factors such as food type and structure, treatment time, and plasma-generating conditions, it is important to establish product-specific treatment parameters.

### 3.6. Effect of Cold Plasma on TBARS Values of Fresh Sea Bass

**Figure 6:**
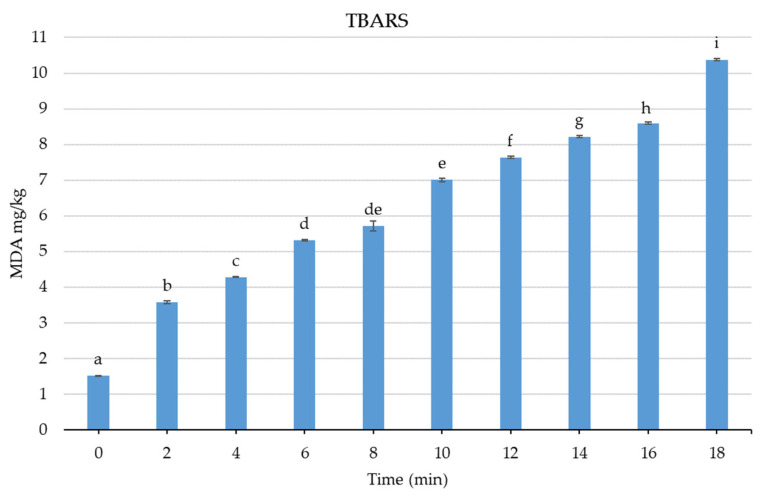
TBARS values of sea bass treated with atmospheric cold plasma (a–i: letters indicate the significant difference between treatments, *p* < 0.05).

TBARS is a widely used index to quantify oxidation levels in fish. It has been reported that plasma treatment may cause lipid peroxidation in fish and create difficulties in maintaining quality [17]. TBARS values of 8 MDA mg/kg [66] and 5–8 MDA mg/kg [67] have been suggested as thresholds for sensory acceptability in fish. In this study, longer exposure times led to significantly higher TBARS levels (*p* < 0.05). The samples processed for 6 min or longer contained TBARS levels above 5 MDA mg/kg, while those processed for 14 min or longer surpassed 8 MDA/kg (Figure 6). In a study aiming to inhibit *Listeria innocua* in Bresaola, a red meat product, via in-package cold plasma, TBARS levels in treated samples increased compared to the control group, with longer treatment times yielding higher values. However, when the plasma application times were short, 20 s or less, TBARS values were low. It was stated that the very lean structure of Bresaola led to low TBARS values, but higher lipid oxidation is likely in fattier foods [38]. Yadav et al. [65] reported that the TBARS amount in ham increased significantly after 3 min of cold plasma application and stated that cold plasma treatment may pose difficulties in terms of the oxidation of meat products. Conversely, no significant change in TBARS levels was observed in pork after 2 min of plasma jet treatment [40]. In this study, seabass was used as the material, and plasma treatment for up to 10 min preserved sensory quality and provided an antimicrobial effect. However, in species with higher fat content, shorter plasma treatments may lead to the TBARS threshold being exceeded more quickly. Therefore, further studies are needed to determine the effects of cold plasma on different aquatic products and identify which species of use would be feasible. Additionally, since prolonged plasma treatments resulted in elevated TBARS levels, combining plasma treatment with natural antioxidants may be recommended to reduce the risk of oxidation associated with the extended plasma exposure needed for pathogen inactivation.

## 4. Conclusions

This study investigated the potential of cold plasma to reduce *S.* Enteritidis and *L. monocytogenes*, which can pose significant food safety risks by contaminating sea bass produced in Türkiye, the EU’s primary supplier. The reductions observed suggest substantial food safety benefits, especially considering the natural contamination levels in fish. The results of this study will assist the seafood industry in applying this emerging technology to enhance microbial safety in fresh fish. According to the findings of this study, 10 min of cold plasma effectively reduces pathogens while preserving the sensory properties of sea bass. If further reduction is desired, cold plasma can be combined with herbal extracts, essential oils, etc. Such combinations may also help to reduce the change in taste and smell. However, regardless of the method used, the impact on sensory properties must be carefully evaluated. The differences in pathogen reduction observed in vitro versus in fish meat highlight the important role of the food matrix in cold plasma treatment. Therefore, the effectiveness of the in vitro treatment must be confirmed in the actual food matrix. Cold plasma technology offers the potential for decontamination while preserving the fresh characteristics of the product, which is difficult to achieve with other decontamination techniques. Additional considerations are necessary for industrial applications, including establishing risk-based treatment parameters for various foods, optimizing equipment design, and evaluating economic costs.

## Figures and Tables

**Table 1:** *S.* Enteritidis and *L. monocytogenes* counts (log CFU/g) on sea bass treated with atmospheric cold plasma (mean ± standard deviation).

Treatment Time (minute)	*L. monocytogenes*	*S*. Enteritidis
0	4.69 ± 0.06 ^a^	4.01 ± 0.01 ^a^
2	4.38 ± 0.04 ^b^	4.01 ± 0.00 ^a^
4	4.37 ± 0.07 ^bc^	3.89 ± 0.02 ^a^
6	4.18 ± 0.11 ^cd^	3.69 ± 0.07 ^ab^
8	4.13 ± 0.05 ^de^	3.33 ± 0.04 ^b^
10	4.10 ± 0.06 ^de^	3.26 ± 0.38 ^bc^
12	4.09 ± 0.07 ^de^	3.09 ± 0.02 ^bc^
14	3.96 ± 0.03 ^ef^	2.92 ± 0.02 ^c^
16	3.89 ± 0.05 ^f^	2.75 ± 0.18 ^c^
18	3.89 ± 0.04 ^f^	2.58 ± 0.27 ^c^

^a–f^ Different letters in each column indicate the significant difference (*p* < 0.05) between treatment times.

**Table 2:** Texture parameters of sea bass treated with atmospheric cold plasma (mean ± standard deviation).

Treatment Time (minute)	Hardness 1	Adhesiveness	Resilience	Hardness 2
0	0.70 ± 0.41 ^a^	0.01 ± 0.00 ^a^	0.34 ± 0.00 ^a^	0.62 ± 0.36 ^a^
2	1.07 ± 0.06 ^a^	0.03 ± 0.00 ^a^	0.34 ± 0.02 ^a^	0.90 ± 0.06 ^a^
4	1.18 ± 0.12 ^a^	0.02 ± 0.01 ^a^	0.36 ± 0.02 ^a^	0.97 ± 0.14 ^a^
6	0.78 ± 0.30 ^a^	0.02 ± 0.01 ^a^	0.31 ± 0.04 ^a^	0.66 ± 0.24 ^a^
8	1.32 ± 0.76 ^a^	0.02 ± 0.01 ^a^	0.34 ± 0.09 ^a^	1.11 ± 0.68 ^a^
10	1.09 ± 0.20 ^a^	0.03 ± 0.01 ^a^	0.26 ± 0.04 ^a^	0.92 ± 0.10 ^a^
12	1.79 ± 0.13 ^a^	0.07 ± 0.04 ^a^	0.30 ± 0.07 ^a^	1.52 ± 0.17 ^a^
14	1.11 ± 0.25 ^a^	0.05 ± 0.02 ^a^	0.31 ± 0.02 ^a^	0.87 ± 0.20 ^a^
16	1.48 ± 0.25 ^a^	0.03 ± 0.01 ^a^	0.30 ± 0.09 ^a^	1.18 ± 0.25 ^a^
18	1.69 ± 0.21 ^a^	0.11 ± 0.03 ^a^	0.23 ± 0.02 ^a^	1.28 ± 0.17 ^a^

^a^ Different letters in the same column indicate significant differences (*p* < 0.05).

## Data Availability

The original contributions presented in the study are included in the article, further inquiries can be directed to the corresponding author.
